# Vitamin C and D supplementation and the severity of COVID-19: A protocol for systematic review and meta-analysis

**DOI:** 10.1097/MD.0000000000026427

**Published:** 2021-07-02

**Authors:** Gislani Acásia da Silva Toscano, Ivani Iasmin de Araújo, Talita Araújo de Souza, Isabelle Ribeiro Barbosa Mirabal, Gilson de Vasconcelos Torres

**Affiliations:** a Postgraduate Program in Health Sciences, Federal University of Rio Grande do Norte, Natal/RN; b Faculty of Health Science of Trairi, Federal University of Rio Grande do Norte, Santa Cruz/RN; c Postgraduate Program in Public Health, Federal University of Rio Grande do Norte, Natal/RN, Brazil.

**Keywords:** Covid-19, severe acute respiratory syndrome Coronavirus, Vitamin C, Vitamin D

## Abstract

**Background:**

The COVID-19 pandemic has rapidly spread to other countries, causing numerous deaths and challenges for organizations and health professionals. Diet and nutrition invariably influence the competence of the immune system and determine the risk and severity of infections. Studies have already been published on the relationships through which vitamins C and D can mitigate the severity of infections such as COVID-19. In this context, this protocol describes a systematic review intended to analyze if vitamin C and D supplementation can reduce the severity of Covid-19.

**Methods:**

This protocol was developed based on the recommendations of PRISMA-P. In order to accomplish the systematic review, we will carry out searches in the PubMed, Web of Science, Scopus, Cochrane, and ScienceDirect databases in the quest for control case studies that analyze the supplementation and evolution of patients with COVID-19. There will be no limitations related to language or publication time. The searches will be carried out by 2 independent researchers who will select the articles, and then the duplicate studies will be removed, while the suitable ones will be selected using the Rayyan QCRI application. In order to assess the risk of bias, we will use the instrument proposed by the National Heart, Lung and Blood Institute. Moreover, we will carry out metaanalyses and subgroup analyses according to the conditions of the included data.

**Results:**

This review will assess the association between vitamin C and D supplementation and the reduction in the severity of COVID-19.

**Conclusion:**

The findings of this systematic review will summarize the latest evidence for the association between vitamin C and D supplementation and COVID-19 through a systematic review and meta-analysis.

**Record of systematic review:**

CRD42021255763.

## 1 Introduction

The 2019 coronavirus disease pandemic (COVID-19), which began in late 2019 and has been spreading around the world on a rapid rise, is caused by infection with Coronavirus 2 (SARS-CoV-2), a member of the coronaviridae family.^[1]^ In the absence of well-documented effective drug treatment,^[2]^ there is great interest in identifying strategies that mitigate the severity of COVID-19.^[3]^ Thus, based on research, it has been proposed that the activation of the vitamin D receptor (VDR) signaling pathway and the use of vitamin C (ascorbic acid, ascorbate) can act as potent antioxidants, decreasing the cytokine/chemokine storm, regulating the renin-angiotensin system, modulating neutrophil activity, and maintaining the integrity of the pulmonary epithelial barrier.^[4,5]^

In a recent research involving 18 adult patients in a COVID-19 ICU who met criteria for acute respiratory distress syndrome (ARDS), 94.4% had undetectable vitamin C levels and 1 patient had low levels.^[6]^ Recently, 2 ecological studies reported inverse correlations between national estimates of vitamin D status and COVID-19 incidence and mortality in European countries^[7,8]^; lower concentrations of circulating 25 (OH) D have also been reported to be associated with susceptibility to SARS-CoV-2^[9]^ infection and the severity of COVID-19 evolution.^[10]^

In this context, we want to analyze of vitamin D and C supplementation in patients with COVID-19 are associated with a reduction in severity (mortality, morbidity, average length of stay, peripheral oxygen capillary saturation, body temperature, and other changes on the World Health Organization's Ordinal Scale for Clinical Improvement (OSCI) for COVID-19.

## 2 Methods and analysis

### 2.1 Protocol and registration

This systematic review was recorded in the International prospective register of Systematic reviews (PROSPERO) on May 20, 2021, under the number CRD42021255763 (Available at: https://www.crd.york.ac.uk/prospero/display_record.php?RecordID=255763).

### 2.2 Selection process

The design and development of this systematic review and meta-analysis will be in accordance with the statement of Preferred Reporting Items for Systematic Reviews and Meta-Analyses (PRISMA-P).^[11]^ Initially, the collection of bibliographic data will be made in the electronic databases: PubMed, Web of Science, Cochrane, Scopus, and Science Direct. In order to carry out the appropriate search in each database, the search strategy will be duly modified for each one and will be carried out by 2 reviewers in a double-blind manner to identify the eligible studies. This pair of independent researchers will carry out the search, and publications considered to be potentially relevant will be included in the review if they meet all the inclusion criteria. Consensus meetings will be held at each stage, if there is no consensus the third reviewer will participate. Cohort studies, sectional-cross, reviews, and qualitative studies will be excluded. The reference list of possible studies included will be selected manually to identify other relevant publications. In case of disagreement, it will be resolved by a third reviewer.

### 2.3 Search strategy

The search strategies will be used from the descriptors and keywords selected for the research, considering the following terms: (“Covid-19” [Mesh] OR “COVID19” OR “COVID-19 pandemic” OR “SARS-CoV- 2-infection” OR “COVID-19 virus disease”) OR (“severe acute respiratory syndrome coronavirus 2”[Mesh] OR “SARS-CoV-2 ” OR “2019 novel coronavirus” OR “COVID-19 virus”) AND (“vitamin C”[Mesh] OR “Ascorbic acid“) AND (”vitamin D" [Mesh]).

The Rayyan QCRI Software,^[12]^ which is used for systematic reviews, will be used to read the titles and abstracts, remove duplicate articles, and read the full texts. For formatting references, we will use the Mendeley Software.^[13]^ Thus, during the process, the title and abstract will be read, then the duplicates will be removed, after which we will read the selected articles in full. If no abstract is provided from this search strategy, the full text can be reviewed and evaluated. If 2 independent researchers do not agree with the inclusion of any study in the review, the third researcher will decide whether or not to include that study ().

**Figure 1:**
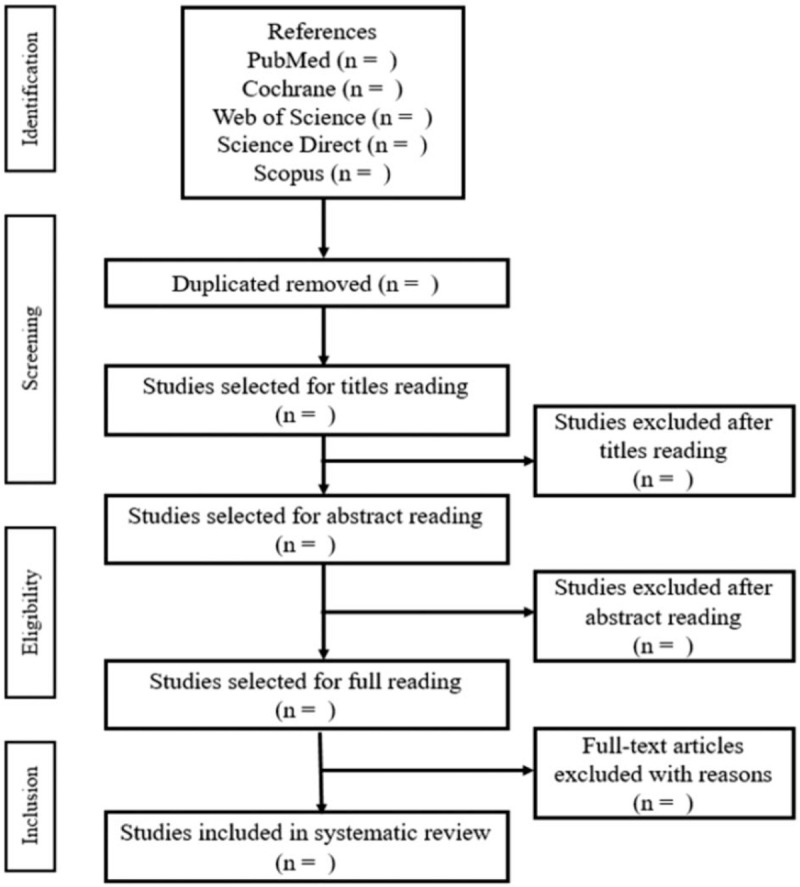
Flow diagram. Adapted from PRISMA-P.

### 2.4 Inclusion criteria

To prepare for this review, we include all control case studies that analyzed vitamin C or D supplementation and evolution of patients with COVID-19, considering the eligibility criteria adopted in the Population of the study, Intervention, Comparison, Result and Study Design (PICOS), according to the details presented in Table 1.

**Table 1:** PICO description.

PICO	Abbreviation	Elements
**P**articipants	P	People with COVID-19
**E**xposition	E	Supplementation oral and / or intravenous vitamin C or D
**C**omparison	C	Supplemented and non-supplemented groups
**O**utcome	O	Less severity after supplementation of vitamin C or D

### 2.5 Exclusion criteria

We will exclude studies according to the following criteria: Studies that do not present the supplementation vitamin C and/or D in their results in patients whit COVID-19; Studies such as cohort, sectional-cross, reviews, and qualitative studies.

### 2.6 Data collection process

The characteristics of the study (author, date of publication, study design, period, type of supplementation, dosage, and route of administration) and study population (place of study, sex, and age group of participants) will be extracted from all included studies. We will identify peer-reviewed publications that include the following criteria: individuals with COVID-19 (participants); people with vitamin C and D supplementation (exposure); people without supplementation (control); Risk greater severity (result). Two reviewers will extract data from all articles independently, and any disagreement will be resolved by a third author.

### 2.7 Risk of bias assessment

To assess the methodological quality of the studies, the articles included will be evaluated and scored according to the quality index for case-control and randomized studies.^[14]^ Each published article will be independently assessed by 2 authors. To resolve any divergences in the assigned scores, a third author will be consulted. The quality tool is a checklist of 12 items on the objectives of the research, recruitment and selection of the study population and sampling, justification for the sample size, the inclusion and exclusion criteria, definitions for case control, simultaneous controls, exposure and assessment measures, blinding and statistical analysis. The ratings on the quality tool are good, reasonable, or bad.

### 2.8 Data synthesis

Results will be expressed as odds ratios (ORs) with 95% confidence intervals (CIs). Fixed-effects or random effects models will be chosen depending on whether there is an absence or presence of heterogeneity between studies. Statistical heterogeneity will be assessed by the *I*^2^ statistic (<25%, no heterogeneity; 25%–50%, moderate heterogeneity; and >50%, strong heterogeneity). When a significant heterogeneity exists across the included studies (*I*^2^ > 50%), a random-effects model will be used for the analysis; otherwise, the fixed-effects model will be used. We will use the Egger funnel plot to assess possible publication bias. All tests will be performed using Review Manager (RevMan version 5.3.0) software and 2-sided *P* value < .05 will be considered statistically significant.

### 2.9 Confidence in cumulative evidence

The GRADE approach will be used to assess the quality of evidence that will be included in this review.

### 2.10 Ethics and dissemination

For the development of this study, approval of ethics and consent is not necessary because it is a systematic review that will use secondary studies.

## 3 Discussion

Nutritional status and diet have important roles in maintaining and recovering health, being even more relevant in the treatment of acute and chronic diseases, as well as in the Ebola outbreak, and can also be applied in the current SARS-CoV-2 (COVID-19) pandemic scenario.^[15]^ Macronutrients, micronutrients, and bioactive compounds are necessary for the integrity of the immune barrier, as well as for ensuring the maintenance of adequate weight, as both malnutrition and obesity are associated with worse outcomes in patients affected by COVID-19, with a higher incidence of hospitalization, longer hospital stay, and higher risk of mortality.^[16]^

In a Spanish study, it was demonstrated that the administration of a high dose of Calcifediol or 25-hydroxyvitamin D3, a major vitamin D metabolite of the endocrine system, significantly reduced the need for intensive care unit (ICU) treatment of patients requiring hospitalization due to COVID-19,^[17]^ while another study carried out in 3 hospitals in China using intravenous doses of vitamin C demonstrated a benefit in oxygenation for critically ill patients with COVID-19.^[5]^

We hope that the completed systematic review will bring results that allow us to understand about vitamin D and C supplementation (dosages and characteristics) in reducing the severity of COVID-19. Furthermore, the results can also assist in the definition of strategies that can be adopted for the control and prevention of COVID-19.

## Author contributions

**Conceptualization:** Gislani Acásia da Silva Toscano, Talita Araújo de Souza.

**Data analysis:** Gislani Acásia da Silva Toscano, Ivani Iasmin de Araújo, Talita Araújo de Souza, Isabelle Ribeiro Barbosa

**Data curation:** Gislani Acásia da Silva Toscano, Ivani Iasmin de Araújo, Talita Araújo de Souza.

**Formal analysis:** Gislani Acásia da Silva Toscano, Isabelle Ribeiro Barbosa.

**Investigation:** Isabelle Ribeiro Barbosa, Gilson de Vasconcelos Torres.

**Methodology:** Gislani Acásia da Silva Toscano, Ivani Iasmin de Araújo, Talita Araújo de Souza, Isabelle Ribeiro Barbosa, Gilson de Vasconcelos Torres

**Project administration:** Gislani Acásia da Silva Toscano, Talita Araújo de Souza, Isabelle Ribeiro Barbosa, Gilson de Vasconcelos Torres

**Reading and Final Revision of the Text:** All.

**Research:** All.

**Resources:** Gilson de Vasconcelos Torres.

**Supervision:** Talita Araújo de Souza, Isabelle Ribeiro Barbosa, Gilson de Vasconcelos Torres.

**Writing – original draft:** Gislani Acásia da Silva Toscano, Ivani Iasmin de Araújo, Talita Araújo de Souza, Gilson de Vasconcelos Torres.

**Writing – review & editing:** Gislani Acásia da Silva Toscano, Ivani Iasmin de Araújo, Talita Araújo de Souza, Isabelle Ribeiro Barbosa, Gilson de Vasconcelos Torres.

**Writing of the scientific paper:** Gislani Acásia da Silva Toscano, Talita Araújo de Souza, Isabelle Ribeiro Barbosa, Gilson de Vasconcelos Torres
